# Prevalence of bruxism and its association with dental malocclusion, condyle shape, and impaction: A cross-sectional study

**DOI:** 10.1097/MD.0000000000050529

**Published:** 2026-09-11

**Authors:** Hassan Ahmed Assiri, Batool Abdullah Asiri, Lama Fahad Almuawi, Raghad Musfer Alahmari, Sarah Amer Alasiri, Shatha Tareq Abumelha, Mohammad Shahul Hameed, Bander Ahmed Assiri

**Affiliations:** a Department of Diagnostic Sciences and Oral Biology and Periodontology, College of Dentistry, King Khalid University, Abha, Saudi Arabia; b General Dentistry, College of Dentistry, King Khalid University, Abha, Saudi Arabia; c Department of Clinical Pharmacy, College of Pharmacy, King Khalid University, Abha, Saudi Arabia.

**Keywords:** bruxism, impacted, malocclusion, mandibular condyle, panoramic, radiography, tooth

## Abstract

This study aimed to investigate the associations between bruxism and impacted mandibular third molars, as well as various malocclusion types and condylar shapes among patients seeking dental care at King Khalid University Dental Hospital. A cross-sectional study design was employed between September 2024 and January 2025. Of the 400 patients screened, 95 had bruxism. Clinical examination and panoramic radiographs were used to assess malocclusion, impacted third molars, and condyle shape. Logistic regression was performed to examine these associations, adjusting for age and sex. The prevalence of bruxism among the sample screened was approximately 23.8%. The median age was 38 years (interquartile range, 28–49 years), and 43.16% of the bruxers were male. Class I malocclusion was the most frequently observed occlusal pattern among bruxers (84.21%), reported descriptively without a between-group comparison. Bruxers had significantly lower odds of third molar impaction (adjusted odds ratio = 0.46; *P* = .013). Condylar shape distributions were similar between groups, with the only exception being a higher frequency of the “angled” morphology among non-bruxers (14.21% vs 4.74%), which we consider an observation requiring confirmation with 3-dimensional imaging. No statistically significant differences in stress, dental attrition, or masticatory muscle complaints were found between those with awake bruxism and those with sleep bruxism. In this clinic-based cohort, Class I was the most frequently observed occlusal relationship among bruxers, consistent with its background prevalence in the general population; no between-group comparison of malocclusion class was performed. Individuals with bruxism had significantly lower odds of having impacted mandibular third molars, and the angled condylar shape was more prevalent among non-bruxers. These findings offer insight into the multifactorial nature of bruxism and underscore the importance of integrating occlusal, radiographic, and behavioral assessments in its management.

## 1. Introduction

The term “bruxism” comes from the Greek word *brychein*, meaning “grinding of the teeth.” Bruxism is defined as an unconscious oral habit of dysfunctional rhythmic pressing, clenching, and grinding of the teeth during movements that are not part of normal masticatory function. It is a complex occlusal parafunctional habit that can be categorized into awake bruxism and sleep bruxism, both of which may have different underlying mechanisms and implications.^[1]^ Bruxism is among the most prevalent and destructive dental parafunctional disorders. Considering the global prevalence of bruxism, Zieliński et al^[2]^ estimated its worldwide prevalence at approximately 21%. Ferrari-Piloni et al^[3]^ highlighted the impact of geographical variations on bruxism prevalence. Recent trends suggest that the prevalence of bruxism is increasing, potentially due to rising stress levels, lifestyle changes, and increased screen time – factors that may contribute to poor sleep quality and altered neurological responses.^[4,5]^ The etiology of bruxism is generally reported to be multifactorial, with no single factor solely responsible for its occurrence.^[6–8]^ Although the precise cause remains unclear, stress and other psychological factors play a major role in its development.^[9,10]^ Bruxism is related to systemic, neurological, and local factors reflecting the patient’s overall quality of life. Several studies have analyzed the causes and consequences of bruxism, considering elements such as stress, neurologic disturbances, drug addiction, occlusal interferences, tooth wear, periodontal condition, and temporomandibular joint (TMJ) dysfunction in various populations around the world.^[6]^ Bruxism has also been suggested to be associated with specific dental characteristics, such as malocclusion, condylar morphology, and impacted mandibular third molars. Some studies propose that dental occlusion may contribute to bruxism, although this role remains controversial.^[11]^ For example, certain researchers indicate that malocclusions – including Angle’s Class II and Class III discrepancies – can trigger parafunctional habits such as bruxism by creating occlusal interferences.^[12]^ Moreover, condylar shape has been studied in relation to TMJ disorders, as variations in condylar morphology might predispose individuals to increased mechanical stress and parafunctional activities.^[13,14]^ Bruxism has been associated with malocclusion and with condylar morphology, with impacted third molars being most commonly researched in reference to purely anatomical and developmental reasons. It is still unclear whether these dentoalveolar and articular factors are related in a bruxing patient group and whether the different levels of third molar impaction reported by various researchers in the Saudi population can be repeated in a larger, general dental hospital-based population. Both impacted third molar teeth and condylar morphology may be used as radiographic measures of a person’s skeletal system (i.e., bones) that reflect prolonged forces of mastication or chewing. Because both features can be recovered from a single panoramic X-ray image taken as part of routine clinical practice and because each feature has been hypothesized to be a correlate of the masticatory forces applied to the jaws over time due to independent theoretical rationales, comparing both features in a common sample of patients yields important information. By describing and examining both features simultaneously, this will determine if they cluster together in bruxers or appear independently. The purpose of this study was to examine and characterize the presence of impacted third molars and condylar morphological differences and their interrelationships in a clinical cohort of Saudi subjects. It was not intended to provide evidence for causal associations between the variables under investigation, nor could the cross-sectional nature of the study provide evidence regarding the directionality of any observed associations. Understanding these associations might help dentists manage patients with bruxism more effectively, strategize preventive treatments, and ultimately improve patients’ quality of life.

## 2. Methodology

### 2.1. Study design and setting

This cross-sectional study was conducted at the King Khalid University Dental Hospital, College of Dentistry (KKUCOD), Abha city, Saudi Arabia, from September 2024 to January 2025. The study protocol was approved by the Scientific Research Committee of KKUCOD (protocol no. IRB/REG/2024-2025/4), ensuring that all procedures met the relevant ethical standards. Written informed consent was obtained from all participants prior to enrollment. The 2018 international consensus definitions were employed to categorize bruxism. Probable bruxism was defined as a combination of a consistent self-report (history of tooth grinding or clenching reported by the patient or their bed partner) and compatible clinical signs (such as attrition facets and/or masticatory muscle tenderness), assessed clinically by 2 calibrated dentists, with any discrepancies resolved by consensus. Instrumental confirmation, such as polysomnography or electromyography, was not utilized in this clinic-based cross-sectional study; hence, our case definition does not align with the criteria for definitive bruxism and may be susceptible to non-differential misclassification.

### 2.2. Sample selection

A total of 400 patients visiting KKUCOD for routine dental treatment or specific complaints were screened for bruxism. A detailed medical and dental history was obtained to assess various clinical parameters, including the presence of dental attrition, self-reported stress level, smoking status, parafunctional habits, and existing dental malocclusions. Eligible patients who attended KKUCOD routinely or with a complaint during the study time frame were invited to enroll and were recruited by consecutive enrollment. This screened cohort represents a clinic-based (convenience-sampled) group and does not represent a randomly selected portion of the general population. As such, the prevalence estimates developed based on this sample should be understood to have limitations when compared to an estimate of the same symptom burden within a community population due to differences in symptom burden and health care utilization behaviors among those who attend a university dental hospital versus the general public. The eligibility criteria included being at least 18 years old and having no syndrome-related condition. Edentulous patients were excluded from the study. The prevalence was estimated from the complete screened cohort (n = 400; 95 individuals identified as bruxers). For analytical comparisons, non-bruxer controls were drawn at random from within the screened non-bruxer pool using 5-year age and sex strata (frequency matching). Random sampling was therefore utilized with respect to the selection of controls from within this clinic-based cohort; however, it was not used with respect to the overall selection of participants into the cohort. Since frequency matching is a method that does not ensure perfect equivalence, we have included additional adjustments for sex and age as part of each model’s adjustment set. To clearly distinguish prevalence estimation from association testing, prevalence data are presented based on the entire screened sample, while associations were assessed within the frequency-matched sub-cohort.

### 2.3. Clinical examination

Two qualified clinicians assessed the patients for signs of bruxism, relying on both self-reported information, such as partners noticing teeth grinding during sleep, and physical signs, such as noticeable tooth wear or tenderness in the jaw muscles or TMJ. Out of 400 patients screened, 95 were diagnosed with bruxism. To create a comparison group, another 95 patients without bruxism were selected from the rest of the participants, matched based on basic demographic characteristics. For all participants, detailed clinical information was collected, including the type of dental malocclusion using Angle’s classification, stress levels, parafunctional habits, and the condition of the TMJ.^[12]^

### 2.4. Radiographic assessment

Panoramic radiographs were taken when clinically necessary using a digital panoramic X-ray unit (Orthophos E/2D; Dentsply Sirona, Bensheim, Germany). Six independent examiners (H.A., L.A., B.A., S.A., S.T., and R.A.) reviewed the images for several parameters, with a 1-month interval between evaluations to reduce bias. Before collecting data on the patients, the raters underwent an initial structured calibration activity, which was conducted by the lead oral and maxillofacial radiologist (H.A.). This involved an educational presentation to provide a clear explanation of Winter’s classification system along with the 5-category system for evaluating condylar morphology. Following that, each rater independently evaluated the same panoramic radiograph from a “training” group of radiographs that were not used in the study. The rater scores were then compared. When there were discrepancies in what the raters had scored, they would review their ratings using reference images to ensure they agreed upon the correct rating. The process continued until there was a satisfactory level of agreement among the raters. It should be noted that the agreement was based on the total radiographic evaluation, rather than each feature individually. Therefore, reliability coefficients cannot be provided for individual features, and this has been identified as one of several limitations. Cohen’s kappa value was calculated to assess inter-examiner reliability for these assessments.^[15]^ The radiographic parameters, including the presence and angulation of mandibular third molar impaction, classified according to Winter’s classification, and the condylar morphology, classified as flat, pointed, angled, round, or irregular, were assessed. Any discrepancies between the examiners were resolved through discussion until a consensus was reached. Regarding the status of third molar impaction on panoramic radiography, it was determined from the first panoramic radiograph acquired at presentation. Our dataset did not include earlier impactions that had resolved or were extracted before this visit; hence, “impaction present” refers specifically to impaction observed on the current radiographic examination.

### 2.5. Sample size calculation

Based on effect size estimates from a previous study and using G*Power software (version 3.1.9.7; Heinrich-Heine-Universität Düsseldorf) with a 95% confidence level (α = 0.05) and 80% power, a minimum sample of 128 participants was required to detect significant associations.^[16]^ Nonetheless, 400 participants were examined to improve the precision of the prevalence estimates and strengthen the validity of the subgroup analyses.

### 2.6. Statistical analysis

Microsoft Excel (Office 2023; Microsoft Corporation) was used to compile data from clinical and radiographic evaluations, and Stata (StataCorp LLC), a web-based interface, was used for the analysis. Since normality testing revealed a nonparametric distribution, the median and interquartile range for age were computed for descriptive statistics. Considering the categorical variables (e.g., malocclusion class and presence of third molar impaction), counts and percentages are described. An equal-proportion null hypothesis chi-square goodness-of-fit test was conducted to evaluate the observed distribution of Angle malocclusion classes within the bruxism group relative to an equal-proportion null. This test characterizes the internal distribution of occlusal classes among bruxers only; it is not a test of association between malocclusion and bruxism, and no such inference is drawn from it. On the other hand, univariate logistic regression was performed to estimate crude odds ratios (ORs) for associations between bruxism and various factors of interest, such as the presence of impacted third molars. Multiple logistic regression multivariate analysis was then conducted to adjust for potential confounders, age and sex, when assessing these associations. Adjustments were made for only 2 factors – age and sex – since those are the 2 variables that were matched by frequency (i.e., the only covariates identified among the 2 populations) as well as being collected from each population. Smoking status and reported stress were collected from the bruxism group only and therefore could not be included in models of between-group differences. Additionally, we intentionally chose not to include adjustments for either dentoalveolar wear or TMJ-related symptomatology since they exist along a causal pathway between bruxism and the outcome measures (dentoalveolar characteristics) being evaluated, and including them in such a model would represent over-adjusting. Statistical significance was set at *P* < .05 with 95% confidence intervals (CIs). The author (B.A.A.) conducted the statistical analysis.

## 3. Results

Of the 400 patients screened in the study, 95 were identified as bruxers, yielding a bruxism prevalence of approximately 23.8%. The median age of the patients was 38 years in both the bruxism and non-bruxism groups (interquartile range, 28–49 years for bruxers and 24–49 years for non-bruxers). The proportion of male patients was 43.16% (41 of 95) in the bruxism group, compared to 27.37% (26 of 95) in the non-bruxism group (Table 1).

**Table 1:** Baseline characteristics of the cohort (bruxism vs non-bruxism groups).

Demographics	Bruxism group (n = 95)	Non-bruxism group (n = 95)
Age, yr – median (IQR)	38 (28–49)	38 (24–49)
Sex, male – n (%)	41 (43.16)	26 (27.37)

IQR = interquartile range, n = number of patients per group.

Just over half of the bruxism patients (53.68%) had sleep bruxism, while 46.32% had awake bruxism. A high prevalence of dental attrition (89.47%) was observed among the bruxers. About half of the bruxers (51.58%) reported experiencing significant psychological stress, and 60% had TMJ-related complaints (such as joint noises or discomfort; Table 2). These clinical features characterize the bruxism cohort and are reported descriptively; since some signs are part of the case definition, they were not interpreted as independent associations.

**Table 2:** Descriptive characteristics among patients in the bruxism group (n = 95).

Characteristic	n (%)
Cigarette smoking, yes	15 (15.79)
Attrition	85 (89.47)
Stress	49 (51.58)
Parafunctional habits	30 (31.59)
Nail biting	4 (4.21)
Lip biting	5 (5.26)
Cheek biting	11 (11.58)
Nail biting and lip biting	2 (2.11)
Nail biting and cheek biting	2 (2.11)
Lip and cheek biting	4 (4.21)
Lip, nail, and cheek biting	2 (2.11)
Type of bruxism	
Sleep bruxism	51 (53.68)
Awake bruxism	44 (46.32)
TMJ disorders	57 (60)
Masticatory muscle pain	6 (6.32)
Disc derangement disorders	20 (21.05)
Inflammatory and degenerative disorders	2 (2.11)
TMJ lateral deviation	11 (11.58)
Multiple TMJ complaints	18 (18.95)
Masticatory muscle complaints	31 (32.63)
Temporalis tenderness	1 (1.05)
Masseter tenderness	1 (1.05)
Medial pterygoid tenderness	4 (4.21)
Lateral pterygoid tenderness	10 (10.53)
Combination of muscle tenderness	15 (15.79)
Sleep disorder	22 (23.16)

All percentages are calculated on n = 95 patients.

n = number of patients in the bruxism group, TMJ = temporomandibular joint.

Within the bruxism group, Class I malocclusion was by far the most common occlusal relationship observed (80 out of 95 bruxers, 84.21%). Class III malocclusion was observed in 8 bruxers (8.42%) and Class II malocclusion in 7 bruxers (7.37%). This distribution differed significantly from an equal-proportions null (goodness-of-fit *P* < .0001), indicating only that the 3 Angle classes were unevenly represented among bruxers. Because Table 3 presents within-group proportions and no between-group comparison, no inference regarding an association between malocclusion class and bruxism can be drawn; the predominance of Class I most plausibly reflects its background prevalence in the general population. When comparing awake bruxism to sleep bruxism within the bruxism group, logistic regression analysis showed no statistically significant differences in the prevalence of stress, attrition, masticatory muscle complaints, or TMJ disorders between the 2 subtypes. This suggests that the timing of bruxism (whether occurring during wakefulness or sleep) alone may not be a key determinant of clinical severity or associated symptoms (Table 4). Bruxism was found to be associated with a lower prevalence of mandibular third molar impaction. In the bruxism group, 31 out of 95 patients (32.63%) had at least 1 impacted mandibular third molar, whereas in the non-bruxism group, 48 out of 95 patients (50.53%) had an impacted third molar. Univariate analysis indicated that bruxers had significantly lower odds of having an impacted third molar compared with non-bruxers (OR = 0.47, 95% CI: 0.26–0.85). After adjusting for age and sex, the association remained significant (adjusted OR = 0.46, 95% CI: 0.24–0.89, *P* = .013), corresponding to approximately a 53% reduction in odds of impaction among bruxers (Table 5).

**Table 3:** Distribution of Angle malocclusion classes within the bruxism group (n = 95; descriptive data; within-group proportions only).

Malocclusion type	Bruxism group – n (%)	*P*-value
Class I	80 (84.21)	<.0001
Class II	7 (7.37)	
Class III	8 (8.42)	

*P*-value from chi-square goodness-of-fit test against an equal-proportion null within the bruxism group. This test does not compare bruxers with non-bruxers and should not be interpreted as evidence of an association between malocclusion class and bruxism.

n = number of patients in the bruxism group.

**Table 4:** Associations between bruxism subtype (awake vs sleep) and clinical factors among bruxers (ORs for awake bruxism relative to sleep bruxism).

Clinical parameter	Crude OR (95% CI)	Adjusted OR (95% CI)
Attrition	2.17 (0.53–8.97)	1.79 (0.41–7.78)
Stress	0.45 (0.19–1.01)	0.50 (0.21–1.16)
Masticatory muscle complaints	0.52 (0.21–1.25)	0.58 (0.23–1.43)
TMJ disorders	0.78 (0.34–1.80)	0.83 (0.36–1.94)

Adjusted for age and sex (multivariate logistic regression). All comparisons between awake and sleep bruxism for these parameters showed no statistically significant difference (*P* > .05 for all).

CI = confidence interval, OR = odds ratio, TMJ = temporomandibular joint.

**Table 5:** Association between bruxism and presence of mandibular third molar impaction (n = 95 per group).

Impaction present – n (%)	Bruxism group	Non-bruxism group	OR (95% CI)	*P*-value
Yes (at least 1 impaction)	31 (32.63%)	48 (50.53%)	0.46 (0.24–0.89)	.013
No	64 (67.37%)	47 (49.47%)	–	–

Adjusted odds ratio from multivariate logistic regression, adjusting for age and sex. The unadjusted OR was 0.47 (95% CI: 0.26–0.85).

CI = confidence interval, n = number of patients per group, OR = odds ratio.

In terms of the type of third molar impaction, there were notable differences between bruxers and non-bruxers (Table 6). Vertical impaction of the mandibular third molar was more frequent among non-bruxers (observed in 50 impactions, 26.3% of non-bruxers) than among bruxers (22 impactions, 11.6% of bruxers), a difference that was statistically significant (*P* = .001). Mesioangular impaction was also more common in non-bruxers (33 cases, 17.4%) compared with those with bruxism (12 cases, 6.3%; *P* = .003). Conversely, distoangular impaction was observed only in the bruxism group (6 cases, 3.2% of bruxers vs 0 cases in non-bruxers; *P* = .02). There was no significant difference in the frequency of horizontal impactions between the 2 groups (4.7% vs 3.2%; *P* = .40). Additionally, with each 1-year increase in patient age, the odds of having an impacted third molar (of any type) decreased by approximately 7%, a finding that was statistically significant (*P* < .001). No significant differences were found in the overall distribution of condylar shapes between bruxers and non-bruxers (Table 7). The proportions of flat, round, pointed, and irregular condyle shapes were similar in both groups (each group had 2 condyles per patient, for a total of 190 condyles in each group). Specifically, flat condyles were seen in 23.16% of condyles in both groups; round condyles in 46% of bruxism patients versus 42% of those without bruxism; pointed condyles in ~12% of both groups; and irregular condyles in 14.21% of bruxism patients versus 8.95% of non-bruxism patients. These differences were not statistically significant (*P* > .1 for each of those shapes). However, the angled condyle shape was significantly more common among non-bruxers’ condyles (27 out of 190, 14.21%) compared with bruxers (9 out of 190, 4.74%; *P* = .002). For the radiographic assessments of impaction type and condyle shape combined, the inter-examiner agreement was substantial: Cohen’s kappa was 0.78, indicating good reliability. This level of agreement, while substantial, reflects some inherent limitations of using panoramic radiographs for detailed morphological assessments.

**Table 6:** Comparison of mandibular third molar impaction types between bruxism and non-bruxism groups (190 mandibular third molar sites assessed per group; 2 per patient).

Impaction type	Bruxism group – n (% of 190 sites)	Non-bruxism group – n (% of 190 sites)	*P*-value
Vertical	22 (11.6)	50 (26.3)	.001
Mesioangular	12 (6.3)	33 (17.4)	.003
Horizontal	9 (4.7)	6 (3.2)	.40
Distoangular	6 (3.2)	0 (0)	.02

Percentages are calculated on 190 mandibular third molar sites per group (2 sites per patient), not on the 95 patients per group. Because each patient contributes 2 sites, these comparisons are presented descriptively.

n = number of patients per group.

**Table 7:** Distribution of condylar process shapes in bruxism versus non-bruxism groups (total condyles examined: n = 190 per group).

Condyle shape	Bruxism group – n (%)	Non-bruxism group – n (%)	*P*-value
Flat	44 (23.16)	44 (23.16)	>.99
Pointed	23 (12.11)	22 (11.58)	.874
Angled	9 (4.74)	27 (14.21)	.002
Round	87 (45.79)	80 (42.11)	.469
Irregular	27 (14.21)	17 (8.95)	.109

Percentages are calculated on 190 condyles per group (2 condyles per patient), not on the 95 patients per group. Because each patient contributes 2 condyles, these comparisons are presented descriptively.

n = number of condyles examined per group.

## 4. Discussion

This study investigated the prevalence of bruxism and its associations with mandibular third molar impaction, types of dental malocclusion, and condylar morphology in a cohort of dental patients in Saudi Arabia. The observed prevalence of bruxism in our sample was 23.8%, which aligns with pooled adult estimates when awake and sleep bruxism are considered together (22%) and may be higher in dental clinic populations versus community samples. It is in accordance with the global prevalence reported in a recent systematic review and meta-analysis by Zieliński et al.^[2]^ Prevalence estimates for bruxism can vary widely in the literature. In Saudi Arabia, studies have reported bruxism prevalence ranging from about 36% up to 66%, depending on the population studied. For instance, Al-Khalifa et al^[17]^ reported a prevalence of 52.7% among military fighter pilots compared with 30.9% in non-pilot controls, with pilots who experienced high occupational stress being 2.6 times more likely to brux than non-pilots. A cross-sectional survey of adults in Riyadh^[18]^ found that 36.4% of participants reported clenching or grinding their teeth. Among university students in the United Arab Emirates, a prevalence of 41.7% was noted, with 38.1% experiencing bruxism during sleep and a subset linking bruxism to headaches.^[19]^ A study of dental students in Jazan (southern Saudi Arabia) reported a bruxism prevalence as high as 66.25%.^[20]^ Differences in study populations, diagnostic criteria, and contributing factors such as stress levels likely account for the broad range of prevalence figures in the literature. In our study, just over half of the bruxism patients had sleep bruxism, while the remainder experienced awake bruxism. The literature indicates that awake bruxism and sleep bruxism are related but distinct conditions: awake bruxism is typically characterized mainly by jaw clenching, whereas sleep bruxism often involves both clenching and grinding of teeth during sleep. Reported prevalence rates for sleep bruxism in adults range between 8% and 15%, and for awake bruxism between 22% and 30%.^[21]^ The split between sleep and awake bruxism in our cohort falls within these expected ranges. The high frequency of dental attrition we observed highlights the mechanical wear associated with chronic grinding,^[8]^ and the psychosocial stress and TMJ complaints reported by our bruxers were correspondingly common. These findings are in accordance with the literature that underscores the important role of psychosocial factors (such as anxiety and stress) in bruxism.^[10]^ Notably, we did not observe any statistically significant differences in the prevalence of stress, attrition, muscle pain, or TMJ disorders between patients with sleep bruxism and those with awake bruxism – indicating that the adverse effects and correlates of bruxism were similar, regardless of when the parafunctional activity occurred. This aligns with findings by Kapagiannidou et al,^[22]^ who also reported no major differences in associated factors between sleep bruxers and awake bruxers, particularly with respect to patterns of tooth wear. Class I malocclusion was the most common occlusal relationship among the bruxers in our study (84% of bruxers), which is unsurprising since Class I is also the most prevalent occlusal relationship in the general population. We did not find a strong association between bruxism and Class II or Class III malocclusions in this cohort. Some studies in the literature have suggested a potential link between bruxism and certain types of malocclusions – for example, an increased tendency for bruxism in individuals with Angle’s Class II or Class III malocclusion.^[12,23,24]^ However, most of the evidence does not support a significant association between bruxism and specific Angle’s classes of malocclusion.^[25–28]^ Our findings are in line with those latter studies, indicating that while malocclusion and bruxism can coexist, 1 is not necessarily predictive of the other. Further research with larger samples would be useful to clarify any subtle relationships between malocclusion severity and bruxism. Interestingly, patients with bruxism in our study had significantly lower odds of having an impacted mandibular third molar compared with non-bruxers. Specifically, bruxers were about half as likely to have an impacted third molar. This finding suggests a potential relationship whereby the chronic mechanical forces generated by bruxism might influence the status of third molar eruption. Because the timing of bruxism status and impaction status were assessed simultaneously, it is possible to infer a directionality (or causation) and exclude noncausal associations. The proposed mechanisms of relationships (occlusal forces – tooth movement/alveolar remodeling) have no empirical support and would require testing through experimental design. Another potential mechanism is confounding. There appears to be a strong association with age; in our study, every year of age reduced the odds of third molar impaction by about 7%. Impaction status was derived from a panoramic radiograph taken upon patient presentation. Therefore, patients who had their third molars removed/extracted prior to this date would not be represented. This means there may be residual group differences based on age, previous extractions, or seeking medical attention, which could explain the association without any biological link. It should be noted, however, that currently there is no direct evidence in the literature establishing that bruxism decreases the likelihood of third molar impaction. The development of third molar impactions is generally thought to depend on factors such as jaw size, tooth size, and the angulation of tooth eruption, rather than on parafunctional habits. While our results raise an interesting question, bruxism’s influence on third molar impaction remains speculative. Bruxism can lead to various dental issues (tooth wear, fractures, and temporomandibular disorders), but its impact on third molar eruption requires further investigation. Additional studies, preferably longitudinal and with detailed imaging, are warranted to explore a possible causal link or mechanism connecting bruxism and the status of third molar eruption/impaction. The analysis of impaction types provided further insight. We found that vertical and mesioangular impactions were significantly more frequent in non-bruxers than bruxers, whereas distoangular impactions were only seen in bruxers (albeit in a small percentage). A recent large-scale retrospective study of 17,760 patients in central Saudi Arabia similarly reported mesioangular impaction as the most common mandibular third molar impaction type, followed by vertical impaction.^[29]^ Generally, the prevalence and type of third molar impactions are determined by anatomical and developmental factors. Bruxism does not directly change the position or angulation of an unerupted third molar, but it could have indirect effects on the surrounding dentition and bone that might, over time, influence impaction outcomes. For example, heavy bruxing forces transmitted through the dentition might alter the interproximal wear or arch spacing, potentially affecting how a third molar emerges. However, given the multifactorial nature of tooth impaction, it is unlikely that bruxism alone plays a major role. Our findings should therefore be interpreted with caution, and more focused research is needed to clarify whether a true association exists between bruxism and the occurrence or type of third molar impactions. Regarding condylar morphology, we did not observe significant differences in most condyle shape categories between bruxers and non-bruxers. The only exception was the “angled” condyle shape, which was more prevalent in the non-bruxers. Overall, our results suggest that bruxism may not drastically alter condylar shape as visible on panoramic radiographs. This contrasts with the findings of a recent study by Mağat and Uzun,^[14]^ which reported a significantly higher prevalence of certain condylar shape variations (described as “bird’s beak” and “diagonal diamond” shapes) among bruxers compared to controls. It should be noted that Mağat and Uzun’s study did not establish a causal relationship – it only indicated a possible correlation. Another recent scoping review by Casazza et al^[30]^ concluded that bruxism could lead to morphological and anatomical changes in the mandibular bone, including the condylar process. Consistent with our findings, Pinheiro et al^[31]^ likewise reported that structural differences of the mandible attributable to possible bruxism are subtle when assessed on panoramic radiographs, reinforcing the view that panoramic imaging has limited sensitivity for detecting bruxism-related morphological change. The discrepancy between our findings and some published reports could be due to differences in sample characteristics, bruxism severity/duration, imaging methods, or definitions of condyle shape categories. It is also possible that changes in condylar morphology from bruxism are subtle or take a long time to manifest and thus might not be easily detected in a cross-sectional radiographic analysis. Importantly, subtle variations in condylar morphology on panoramic radiography are prone to misclassification; any shape-specific differences should be regarded as hypothesis-generating and confirmed through cone-beam computed tomography (CBCT) or magnetic resonance imaging (MRI)-based studies. Further research, potentially incorporating more advanced imaging modalities (such as CBCT or MRI) and longitudinal follow-up, is necessary to better understand if and how bruxism affects the TMJ structures.

### 4.1. Limitations

There are several limitations of this study. First, participants were consecutively recruited from a single university dental hospital; as such, the cohort is clinic-based instead of population-based, and patients attending such centers differ systematically from community samples by symptom burden and care-seeking behavior, which typically inflates prevalence estimates. Neither the prevalence figure nor the associations reported here should be generalized to the larger Saudi populace, and multicenter repetition, including primary care and community settings, will be required. Second, the cross-sectional design captures exposure and outcome at a single time point and cannot establish temporality; all associations reported are correlational. Third, adjustment was limited to age and sex since smoking status, self-reported stress, and Angle malocclusion class were ascertained within the bruxism group only and could not be entered into between-group models. Residual confounding by these and other unmeasured factors (including bruxism duration/severity, orthodontic and extraction history, and cumulative occlusal wear) therefore cannot be excluded. Furthermore, malocclusion class could not be formally compared between groups. Fourth, bruxism was classified as probable according to the 2018 international consensus without instrumental confirmation by polysomnography or electromyography. Non-differential misclassification of exposure is therefore likely and would be expected to bias the observed associations toward the null. Fifth, panoramic radiographs are 2-dimensional projections subject to magnification, superimposition, and position error and are not the reference standard for condylar assessment. Shape-specific findings should therefore be regarded as hypothesis-generating pending confirmation with CBCT or MRI. Sixth, agreement between examiners was assessed for the combined radiotherapeutic assessment instead of separately for impaction classification and condylar morphology, so feature-specific reliability cannot be reported. Prospective collection of an identical variable set in both groups is a priority for future work.

## 5. Conclusion

This study was based on a clinic-based cross-sectional sample. Approximately 24% of participants were found to have probable bruxism. The most common occlusal relation (Class I) seen among bruxers was also the most common in the overall population, which supports its background prevalence. Bruxers had significantly reduced odds of having an impacted mandibular third molar as compared to frequency-matched non-bruxer patients. Non-bruxer patients more commonly exhibited “angled” condylar morphology. These data represent associations (not causal relationships), derived from observations made once in 1 location. There is no basis for inferring causality or prediction regarding whether bruxism causes, prevents, or alters the likelihood of third molar impactions or the condylar morphology. However, these data do provide support for a broader perspective that assessing bruxism will benefit by combining assessments of psychological/behavioral, occlusal, and radiographic factors rather than isolating each factor. Further longitudinal studies incorporating instrumental confirmation of bruxism and 3D imaging would be necessary prior to drawing any implications for clinical practice.

## Acknowledgments

The authors would like to acknowledge the personnel at the King Khalid University Dental Hospital for their professional cooperation throughout the project. No individual is named in this section, and no individual permissions were therefore required. The authors extend their appreciation to the Deanship of Research and Graduate Studies at King Khalid University for funding this work through the Large Research Groups Program under grant number RGP2/494/47.

## Author contributions

**Conceptualization:** Hassan Ahmed Assiri.

**Data curation:** Bander Ahmed Assiri.

**Formal analysis:** Raghad Musfer Alahmari.

**Funding acquisition:** Mohammad Shahul Hameed.

**Investigation:** Batool Abdullah Asiri, Lama Fahad Almuawi, Sarah Amer Alasiri.

**Methodology:** Sarah Amer Alasiri.

**Project administration:** Shatha Tareq Abumelha.

**Resources:** Batool Abdullah Asiri.

**Validation:** Shatha Tareq Abumelha.

**Visualization:** Mohammad Shahul Hameed.

**Writing – review & editing:** Hassan Ahmed Assiri.

**Writing – original draft:** Raghad Musfer Alahmari.
